# Structural Biology by NMR: Structure, Dynamics, and Interactions

**DOI:** 10.1371/journal.pcbi.1000168

**Published:** 2008-09-26

**Authors:** Phineus R. L. Markwick, Thérèse Malliavin, Michael Nilges

**Affiliations:** Institut Pasteur, Département de Biologie Structurale et Chimie, Unité de Bio-Informatique Structurale, CNRS URA 2185, Paris, France; National Center for Biotechnology Information (NCBI), United States of America

## Abstract

The function of bio-macromolecules is determined by both their 3D structure and conformational dynamics. These molecules are inherently flexible systems displaying a broad range of dynamics on time-scales from picoseconds to seconds. Nuclear Magnetic Resonance (NMR) spectroscopy has emerged as the method of choice for studying both protein structure and dynamics in solution. Typically, NMR experiments are sensitive both to structural features and to dynamics, and hence the measured data contain information on both. Despite major progress in both experimental approaches and computational methods, obtaining a consistent view of structure and dynamics from experimental NMR data remains a challenge. Molecular dynamics simulations have emerged as an indispensable tool in the analysis of NMR data.

## Introduction

The function of bio-macromolecules is determined by both their 3D structure and conformational dynamics. These molecules are inherently flexible systems displaying a broad range of dynamics on time-scales from picoseconds to seconds. Nuclear Magnetic Resonance (NMR) spectroscopy has emerged as the method of choice for studying both protein structure and dynamics in solution. The principle behind NMR-based structure determination is to obtain a set of empirical structural parameters, such as inter-atomic distances and dihedral angles, which are implemented in the form of restraints in a molecular modeling algorithm to obtain a representation of the 3D structure of the bio-molecule. The empirical data are acquired from the study of different NMR-based “relaxation channels” or mechanisms that are sensitive to both molecular structure and dynamics. Several recent reviews discuss methods for NMR protein structure calculation [1]–[5]. The present review focuses on NMR methods that both determine and assess biomolecular structure or dynamics in the most objective way and focuses on the challenges involved in obtaining a consistent view of structure and dynamics from the available experimental NMR data.

## 3D Structure Determination from NMR Data

**Figure 1:**
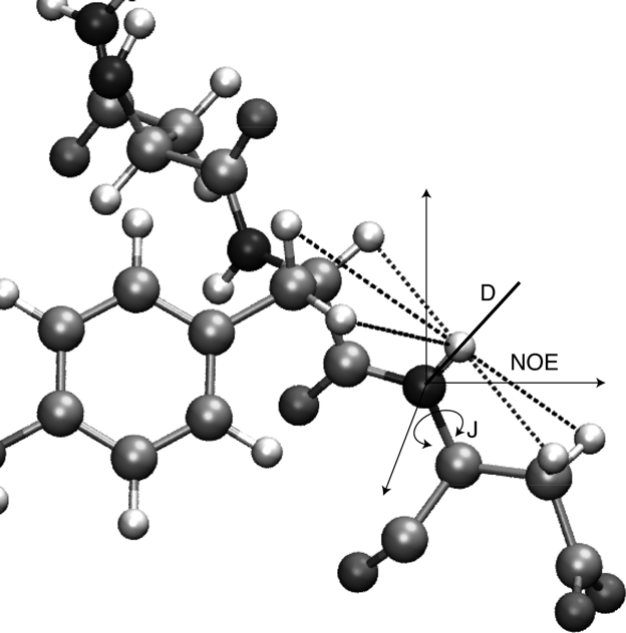
Illustration of structural data that can be obtained from NMR experiments, with the example of a backbone NH group. Dashed line: short inter-proton distances based on NOE measurements; arrow: torsion angle from scalar couplings (J); heavy line: orientation of a bond vector in a coordinate frame rigidly attached to the molecule from residual dipolar couplings (D).

The NOE (Nuclear Overhauser Effect), a through-space relaxation mechanism involving the transfer of tranverse magnetisation between local spin-active nuclei, provides information about average inter-atomic distances up to about 6 Å [6], and remains the most important data for structure determination by NMR. More long-range–distance information can be obtained from paramagnetic relaxation [7]. Distance information is supplemented by the measurement of torsion angles from through-bond scalar J-couplings. Residual Dipolar Couplings (RDCs) [8],[9] provide additional structural information concerning the orientation of inter-atomic vectors with respect to a reference frame. The principal data types are illustrated in Figure 1. The primary data of any NMR experiments, the chemical shifts (the resonating frequencies of the nuclei), depend on the local magnetic field and hence reflect the local molecular environment [10]. Combined with a library of short fragments of known 3D structure, the chemical shift alone can be sufficient to determine the 3D structure [11],[12].

In general, structure determination from NMR relies on data from liquid samples, and it has only recently been demonstrated that structure determination of proteins by solid state NMR is feasible [13],[14]. This has potential applications for molecules that neither are soluble nor form three-dimensional crystals easily, such as membrane proteins.

The experimental information on structure and dynamics is intricately mixed. For example, the NOE depends on the distance (a structural parameter, Figure 1) and on the angular fluctuation, which, if the distance is known and fixed, can be used to characterise local dynamics. Structural parameters extracted from NMR data are therefore rather approximate in nature; for the NOE derived distances, the error may be on the order of 2 Å. Due to the difficulties in the interpretation of the data, structure determination and analysis of dynamical information are in general performed independently.

### 

#### Increasing speed and reliability of NMR structure determination

NMR structure determination still presents some considerable challenges: the method is limited to systems of relatively small molecular mass, data collection times are long, data analysis remains a lengthy procedure, and it is difficult to evaluate the quality of the final structures. These issues are particularly apparent when using NMR in structural genomics projects [15], and advances have been made in all areas. The calculation of a structure itself has become extremely rapid [16], and new labelling methods [17] have significantly improved both spectral quality and automated analysis, whilst rigourous standards and data formats afford compatibilty of different software packages [18].

The most significant advances in efficiency have been gained through automation of data analysis and structure calculation [16], [19]–[21]. An analysis of the network of NOEs reduces the number of possible assignments of an NOE prior to the structure calculation [22],[23] and leads to a considerable improvement in algorithm performance. Such protocols require prior knowledge of the frequencies of all nuclei (^1^H, ^13^C, ^15^N). Automated chemical shift assignment has become more reliable [24], and with good data may allow fully automatic structure calculation [25].

An attractive idea is to dispense completely with the chemical shift assignment and to calculate “clouds” of (covalently unconnected) protons directly from the NOE data. Ambiguities in the NOE data make this a difficult task, even for the most successful implementations [26]. Results with a partial implementation of this idea limited to missing chemical shift assignments in automated structure calculations [27] are encouraging. RDCs allow one to obtain the fold of a protein [28]–[30], and in combination with ab initio fold prediction software and subsequent filtering with NMR data, allow for rapid automated assignment and fast fold determination [31].

When implemented in combination with other data, such as intermolecular NOEs, RDCs are also extremely useful in the determination of molecular complexes [32] or multi-domain proteins. Similarly, the combination of NMR and small angle X-ray scattering (SAXS) methods can be applied [33]. Chemical shift variations may also be employed in the form of additional inter-molecular distance restraints [34]. Using appropriate labelling techniques, the application of NMR methods can be extended to the characterisation of molecular interactions in very large molecular assemblies [35],[36], and, importantly, NMR allows the study of transient interactions and of low-affinity complexes [37],[38].

Assessment of the quality of data and structures is of utmost importance, particularly when automation protocols are employed [39]. Validation tools can assess the consistency of the data directly by information theoretical methods [40]. The Bayesian structure calculation method discussed below inherently validates both structures and data [41],[42]. New NMR quality assessments based on statistical methods [43] provide a global measure of the agreement of the calculated structures with the NOESY peak lists. The importance of using state-of-the-art structure calculation protocols and adapted force fields [44] has been demonstrated by a systematic re-calculation of a large number of structures [45].

#### Towards objective NMR structures

In order to calculate a 3D structure from data, it is necessary to use a model or a theory to calculate the data from the atomic coordinates. Incompleteness of the data, experimental errors, and approximations in the theory make it, strictly speaking, impossible to calculate the structure directly from the experimental observables. A further problem of the direct approach is that most theories contain unmeasurable parameters that need to be estimated before the calculation.

The standard approach to structure determination is to set up a so-called hybrid energy *E_hybrid_* = *E_phys_*+*w_data_ E_data_* [46],[47], where the non-physical term *E_data_* assesses the consistency between the experimental data and the coordinates, and the physical energy *E_phys_* complements the experimental values with information known a priori, such as covalent bond lengths [47]. Conceptual problems with this approach are circumvented by introducing ad hoc assumptions and modifications, for example, by introducing bounds on NOE-based distances [6] rather than using the specific experimental values.

Repeated minimisation with the same empirical data results in structural divergence, which reflects the incompleteness of the data. The divergence is used as an ad hoc measure of the uncertainty of the solution, its “precision” [48],[49]. Incorrectly, it is also sometimes regarded as a measure of the extent of molecular motion. Coordinates and their precision are influenced by many factors, such as the choice of auxiliary parameters and the limitations in the minimisation algorithm. In consequence, structure determination by NMR is often perceived as less objective than X-ray crystallography. A recent analysis of errors in published NMR structures [50] highlights the danger of subjective elements in structure determination procedures.

In contrast to the standard approach, the Inferential Structure Determination (ISD) method considers structure determination as an “inference problem,” which is appropriate since the empirical data are incomplete and uncertain [41]. It differs from the standard approach both in the way it uses the data and in the way it generates and evaluates the resulting structures. Data enters the calculation as close to raw data as is feasible; the theory to calculate data from the structure and an error model is explicitly formulated within the formalism. The error model accounts for deviations between calculated and measured observables. A force field *E_phys_* provides the prior knowledge concerning biomolecular structural parameters. These two terms resemble the two terms in the hybrid energy function introduced above. In contrast, however, probability calculus is used to assign and rank a “posterior probability” to every conformation. All unknown parameters are estimated during the calculation, making ISD a method without free parameters. This includes the unknown error of the empirical measurements, which is related to the weight of the experimental data [51].

Inferential structure determination consists of exploring the conformational space to obtain a probability distribution for the structural ensemble. Since systematic exploration of all possible structures is unfeasible, ISD uses an appropriate sampling strategy (replica-exchange Monte Carlo [52]) that visits regions of conformational space with a frequency proportional to the posterior probability. Compared to conventional structure calculation techniques, a Bayesian approach is computationally more challenging, because distributions of structures need to be explored.

The importance of the approach does not lie primarily in improved convergence for sparse data but in the fact that it puts calculation from experimental data on a sound theoretical basis: no ad hoc rules, nor empirical estimations of parameters, are necessary. It is in principle applicable to the interpretation of all forms of experimental data, and also for other applications such as comparative modelling.

## Probing Structural Dynamics by NMR

The most severe approximation to structure determination is the general assumption that the experimental data can be represented by a single structure, neglecting the effects of internal dynamics. The ISD approach deals with statistical uncertainties in a rigourous manner, maintaining, however, this single copy model. Any ensemble generated by ISD or repeated minimisation cannot represent true dynamics, but only lack of information. It is therefore not meaningful to try to optimise the precision of the ensemble to some expected (or measured) dynamical property.

Attempts to go beyond the single structure approximation by ensemble or trajectory averaging have a long history [53]–[55]. In these studies, the molecular dynamics (MD) force field is not only used to complement the absence of structural information, but also the motion observed in the MD trajectory is employed as a model to explain dynamical features. Adding a pseudo-potential for the experimental data in an MD simulation perturbs the dynamics in a non-predictable manner, making a detailed analysis of the resulting trajectories difficult. For example, the ensemble of structures generated in such a way cannot be expected to have the correct relative free energy weighting for each ensemble member.

**Figure 2:**
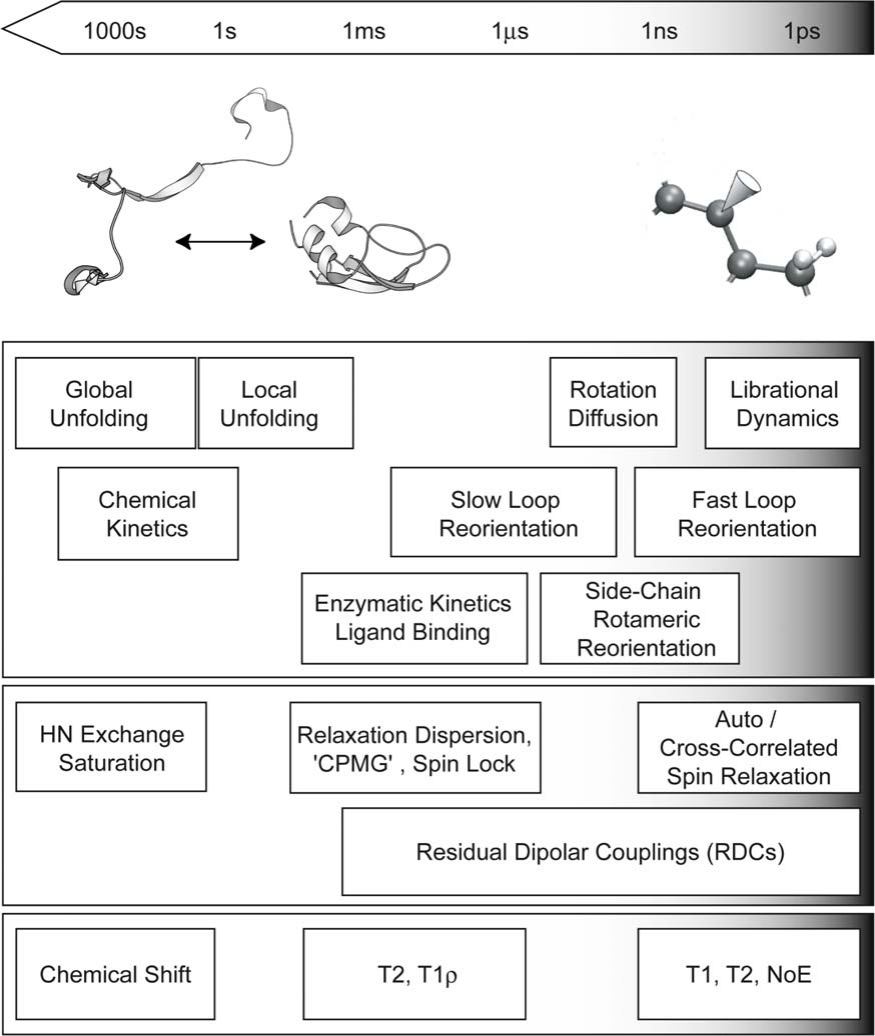
Time-scales, molecular motions (first panel), NMR experiments (second panel), and NMR parameters (bottom panel). Molecular motions at a particular time-scale can be probed by analysing the NMR observables in the bottom panel by using the appropriate NMR experiments (see text). Indicated are the extreme cases, folding/unfolding (which can be observed with hydrogen exchange saturation via the chemical shift) and fast local dynamics (observed with spin relaxation experiments through measurements of the relaxation times *T*_1_, *T*_2_, and the NOE between the amide nitrogen and the amide proton). The cone indicates, in a qualitative way, the spatial extension of the motion described by the N-H group.

NMR is an ideal tool for probing dynamics occurring across a broad hierarchy of time-scales. Difficulties occur in the specific interpretation and quantification of the dynamic processes being observed. Raw experimental NMR data can provide detailed information concerning dynamically active regions in the system occuring on a particular time-scale (see Figure 2). However, this information is encoded in a complex manner and does not directly provide specific information about the molecular motions. To this end, experimental NMR data is complemented by the use of geometric models and increasingly by MD simulation to characterize at an atomistic level local dynamic processes and complex structural transitions.

### 

#### Fast time-scale local motions

Precise information about local dynamics on pico- to nano-second time-scales can be obtained by spin relaxation measurements. Spin relaxation has been used to study fast time-scale dynamics in proteins for several decades, but the study of fast motions remains a rapidly developing and exciting field employing an increasing variety of experimental and theoretical methods. The importance of fast time-scale dynamics is often under-estimated: fast time-scale motions act to stabilize the protein in its folded state, and their presence is a necessary pre-requisite for slower time-scale dynamics involving large-scale collective motions [56].

The traditional “model-free” [57] approach describes local internal motions using two parameters (an order parameter characterising spatial restriction of the motion, and a relaxation time) without making reference to a specific motional model. On the other hand, numerous analytical models have been developed to describe fast time-scale local dynamic fluctuations [58]–[60]. One of the most popular anisotropic models is the 3D-Gaussian Axial Fluctuation (GAF) model [61] based on the observation of peptide plane motions extracted from an MD trajectory. Alternative approaches to interpreting spin relaxation make use of the strong relationship between structure and local dynamics [62],[63] to rapidly predict ^15^N order parameters from a known structure.

In order to provide a more complete description of fast time-scale dynamics, numerous experiments have been developed to obtain order parameters complementary to the N-H vector in both the backbone and side-chains [64]. Cross-correlated relaxation (CCR), which arises from the interference of two relaxation mechanisms such as the chemical shift anisotropy (CSA) and dipole–dipole interaction, has emerged as a powerful tool to study local anisotropic dynamics. For example, by combining a CSA model based on density functional theory (DFT) calculations [65] with MD simulation, it was possible to reproduce to a high degree of accuracy a complete set of CO CSA/DD CCR rates [66]. In the framework of the 3D-GAF model, it was shown that local anisotropic motions can be accurately estimated from a single CO/N-H CCR rate.

The study of local dynamic fluctuations using MD simulation is now routine. Experimentally determined order parameters are regularly used to gauge the accuracy of MD simulations [67]. Continued research in the area of force-field development [68] has resulted in a marked improvement in the prediction of order parameters [69]. The inclusion of polarization and quantum effects of the atomic nuclei in the next generation of force-fields will no doubt lead to further improvement. However, discrepancies between experimental and simulated order parameters may not be solely due to inadequacies in the force-fields, but to incomplete conformational sampling [70].

Despite the maturity of the field, many issues remain unresolved. For example, different models of molecular motion are equally capable of reproducing the experimental results [71]. Many long-held assumptions concerning the local molecular geometry of the peptide plane, and in particular the position of the amino-proton are being revisited. Also, the generally accepted idea that fast internal motion and overall molecular tumbling can be treated independently has been brought into question [72].

#### Going beyond the nano-second limit

Many biologically important processes, such as enzyme catalysis, signal transduction, ligand binding, and allosteric regulation occur on the micro- to milli-second time-scale. Despite their obvious importance, the study of these slow motions remains a challenge to both experimentalists and theoreticians alike. The study of dynamics at these longer time-scales are centred mostly on relaxation dispersion and RDC measurements.

In the presence of a suitable alignment medium, RDCs are averages over all orientations of the magnetic dipolar interaction tensor up to a time-scale defined by the inverse of the alignment-induced coupling. This makes them sensitive to dynamic processes up to several milli-seconds. That RDCs can probe dynamics on extended time-scales was recognised early [8]. However, even today there are conflicting views concerning the sensitivity of RDCs to slow time-scale motions and the ability to separate the contributions to RDCs arising from structural and dynamic properties of the system. Thus, several studies on model protein systems [73],[74] have concluded that a single copy representation of the molecule is in general sufficient to explain the data, and that only a small subset of residues exhibit large amplitude fluctuations on slower time-scales. In contrast to this, simultaneous structure–dynamics determination approaches have suggested the presence of significant slow time-scale molecular motions. Independent studies performed on ubiquitin using model-free approaches [75]–[77] showed an effective homogeneous distribution of long time-scale dynamics across the molecule. A 3D-GAF–based RDC analysis of the protein GB3 suggested a heterogeneous distribution of highly anisotropic long time-scale dynamics [78],[79]. In part, the discrepancy between different analyses can be ascribed to the very small number of systems studied in detail to date, and no general trends can be expected as yet. However, considering the fact that the two proteins studied in most detail (GB3 and ubiquitin) show a similar fold, it is surprising that the observed distribution of slow motions appear to be so different.

RDCs provide a detailed quantitative view of the time- and ensemble-averaged protein structure and the amplitude and direction of slow time-scale motions; recently, RDCs have also been obtained for excited protein states [80]. Considering the wealth of structural and dynamic information encoded in experimental RDC data as discussed previously in this Review, the observation of RDCs in these low-populated states may well provide a completely new direction for the study of long time-scale dynamics in proteins.

The characterisation of motion by relaxation dispersion involves measuring the excess transverse relaxation rate caused by the exchange of nuclei between different conformational states or sites with different characteristic chemical shifts. Recent methodological advances in experimental techniques have extended both the time-scale of observable dynamic processes [81] and the sensitivity [82] of the experiments to exchange processes involving enzyme catalysis [83]–[85], regulation [86], and ligand binding [87],[88]. Lewis Kay and co-workers have developed an entire suite of relaxation dispersion experiments allowing the study of “invisible,” low-populated excited states in proteins probing exchange processes in both the backbone and side-chains [87], [89]–[91].

Relaxation dispersion experiments provide information concerning the location of dynamically active sites in a molecule and the exchange rates between the different conformational states as well as their relative free energies (and thus their populations). Unfortunately, relaxation dispersion experiments do not provide any direct structural information about the different conformational states, and a structural model of the dynamic processes observed is difficult to extract. This makes it necessary to combine the information with other experiments [92] or MD simulations.

Despite the continual increase in both available computational power and efficiency of contemporary algorithms, the simulation of slow motions in proteins involving stochastic transitions over large energy barriers on the rugged and highly structured potential energy surface remains a challenging and active field of research. Considerable progress has been made in the development of new methods to sample the conformational space of proteins more efficiently such as conformational flooding [93], accelerated MD [94], and many others reviewed recently [95]. “Biased potential” MD simulations have successfully identifed large-scale slow collective motions in proteins [96],[97]. A 0.2 µs “brute-force” MD simulation of ubiquitin showed considerable dynamics occurring on time-scales beyond those probed by spin-relaxation measurements [98], and, very recently, accelerated MD simulations of the GB3 domain reliably reproduced RDC-based order parameters [70]. In light of these early successes, the study of long time-scale dynamics using a combination of MD simulation and experimental NMR holds great promise for the future.

## Conclusions and Outlook

The fundamental challenge to NMR remains to combine and reconcile all the available information, both structural and dynamic, into a complete, and therefore intrinsically more accurate, representation of the conformational space sampled by biomolecular systems, with the aim of resolving the relationship between structure, dynamics, and function.

One of the most interesting aspects of NMR is that it is not limited to the study of highly structured systems, and an exciting new application of NMR-based experiments has emerged in the field of natively unstructured proteins. Fully or partially natively unstructured proteins make up a substantial part of protein sequences coded in eukaryotic genomes [99], and they play a key role in some of the most important biological processes and degenerative pathology. It is possible to measure small but finite RDCs from natively unstructured or unfolded proteins [100]. The interpretation of these RDCs is rather complex, since a single structure is certainly no longer appropriate in this case; rather a large ensemble of interchanging structures is required to fully describe the conformational behavior of the system, generated by random sampling [101],[102] or by accelerated MD [103].

The close connection between experimental NMR, molecular modeling, and MD simulation has a long history. Molecular modeling approaches and simulations are necessary to interpret the data, whilst NMR experiments serve to act as a guide for the improvement of force fields. The study of long time-scale dynamics and unstructured proteins provides new and exciting challenges to both theoreticians and experimentalists.
